# VAL-U: Psychometric properties of a Values and Civic Attitudes Scale for University Students’ Service-Learning

**DOI:** 10.1186/s41155-021-00206-8

**Published:** 2022-01-04

**Authors:** Yolanda Ruiz-Ordóñez, Amparo Salcedo-Mateu, Ángel Turbi, Carlos Novella, Carmen Moret-Tatay

**Affiliations:** 1 Universidad Católica de Valencia San Vicente Mártir, San Agustín 3, Esc. A, Entresuelo 1, 46002 València, Spain; 2 Dipartimento di Neuroscienze Salute Mentale e Organi di Senso (NESMOS), La Sapienza University of Rome, 00185 Rome, Italy

**Keywords:** Service-learning; Values; Civic attitudes; Psychometric properties

## Abstract

The service-learning disciplines can offer a unique opportunity for civic development in university students, as there is a large body of research that links it to values and civic attitudes including a vast number of ecological issues and citizen variables. Moreover, one should bear in mind that these students are future generations that will face many pressing social and environmental issues. Given the need to develop instruments to measure the impact of a service-learning methodology in university students’ values as well as civic attitudes, VAL-U is proposed. As the university can be considered a learning step prior to the professional field, the main objective of this study was to analyse the internal consistency and factor structure of the proposed VAL-U scale in the Spanish population. The scale confirmed acceptable psychometric properties. Furthermore, the results have shown high reliability and optimal goodness of fit. Promising results are offered to employ VAL-U as a valuable tool for assessing Values and Civic Attitudes Scale for Spanish-speaking University Students’ Service-Learning.

## Introduction

The trajectory that learning has had in the university in recent years has proven to be a very complex process when it comes to acquiring academic skills (Dias & Soares, 2018). To this end, the service-learning (SL) disciplines can offer a unique opportunity for the civic development of university students. It is important to remember that future generations will face many pressing social and environmental issues of a citizen nature (Burton, 2018). In this way, collaboration among other variables might improve the skills to make democratic decisions together. Moreover, it could be also considered a benefit to improve different elements of the curriculum, by ensuring our students have endless real opportunities through this pedagogy. All these variables have been combined with different actions aimed at improving real-life aspects that need active emotional participation (Sanders et al., 2016). In this regard, acquiring values while implementing academic competencies, promotes social inclusion, activates networks of commitment, sustainability and inclusive awareness (Hébert & Hauf, 2015; Martínez-Campillo et al., 2019).

There is a large body of research that links values with SL experiences (Dienhart et al., 2016; Ruiz-Ordóñez et al., 2020). Therefore, it is of interest to develop tools to measure these associations, and more precisely, the acquisition of values when applying the service-learning methodology. Most of these values have evolved throughout history adapting themselves to everyday reality and a society in continuous globalisation although the essence of their meaning is based on the immediate support or help that a person or group can offer in each emergency. University is a principal agent for addressing the sustainable development goals, where immediate social connotation about the terms is raised above any political, cultural, or religious difference. Moreover, SL might be a powerful tool for social transformation, although little research has been carried out on its academic impact in the Higher Education context (Norman, 2018). Although there is a universal consensus in the acquisition of values, cultural preferences always stand out. According to these authors, the main components of civic values would relate to a sense of group and social support, as well as commitment and solidarity.

On the other hand, human values play a crucial role in human actions, influencing several aspects of life. It has been commonly argued that values should be taken into consideration as drivers of well-being. Moreover, some literature has suggested a relationship between values and well-being which sheds light on how extrinsic values such as security, power and tradition are associated with lower life satisfaction. Moreover, human values have been linked to prosocial behaviour. More precisely, this is understood to mean that any behaviours that a person willingly does whose objective is the benefit of others is known as prosocial behaviour (Caprara et al., 2014). With regards to achievements and their effect on well-being, a piece of research (Gerbino et al., 2018) found in two different studies with Italian adolescents that prosocial behaviour is a strong predictor for academic achievement in the short and long term, which in turn has been related to well-being. Thus, most of the values proposed and related to SL in the literature are sorted in terms of responsibility, commitment, solidarity, respect, and dialogue. The value of responsibility can be considered a characteristic related to morality, which leads us to accept the consequences of our actions. Moreover, within the University environment, the students would fulfil the commitment to put into practice their personal values by taking committed actions in daily life, and by act responsibly and properly. Commitment is also inherent to a society, where this term is the indispensable element for the community to progress as a result of their involvement as educational agents (e.g., managers, teachers, families, among others) and citizen institutions that work for the common good (Huppert, 2009). Furthermore, respect is the basis for moving towards a more equitable and humane society. It is a concept rooted in social action, where we include respect the other, the dignity of the person and the absolute value of every human being (Jaeger & Thornton, 2006).

SL promotes the necessary conditions for all students to feel, and exercise based on understanding, dialogue, respect for diversity, dignity, and values. But SL methodology is also ideal for supporting both the school and the family in the development of civic values. On the other hand, one should find solidarity in the literature, as a moral virtue and social attitude. It consists of fighting against the structural causes of poverty, inequality, lack of work, land and housing, denial of social and employment rights (Delano-Oriaran et al., 2018). According to Prentice (2007) service-learning can rise students’ civic engagement, when civic engagement is defined as more than just political action.

Even if different studies have found human values, and consequently their components, to be an important tool in life since they are intimately related to well-being, the scales developed in the field are not so common. For example, an interesting tool was developed by Weber et al. (2004). On the other hand, current approaches seem to be interested in the cultural dimension underlying this field (Chow et al., 2020). Given the need to develop instruments to measure the impact of the SL methodology on student values, as well as civic attitudes, current research aims to develop a scale to shed light on values and civic attitudes related to University Students’ Service-Learning. Therefore, the main objective of this study was to analyse the consistency and internal structure factor of the proposed VAL-U scale. To date this has been a challenge in the Spanish population, since many tools are in English, but not for this population.

## Materials and methods

### Participants

To analyse the factor structure of the proposed VAL-U scale, two independent samples were selected. A convenience sampling method was used for both cases. Participants collaborated voluntarily and received no compensation. Firstly, a sample of 162 Spanish undergraduate psychology students (79% were women and 21% men) with an age range between 18 and 31 years, M= 21.39, SD= 2.48, participated in the exploratory factor analysis. Secondly, an independent sample of 228 Spanish undergraduate psychology students participated in the confirmatory factor analysis (71.9% women and 28.1% men). They depicted an age range between 18 and 41 years, M= 20.53, SD= 3.63.

A third, and final sample of 20 students (80% women and 20% men) was included to examine retest reliability. These participants were recruited from the second sample and they were evaluated in two different moments. The follow-up was carried out 5 months after the previous measure. They described an age range between 19 and 29 years, M= 21.65, SD= 2.39. Even if higher samples are commonly recommended, GPower (Erdfelder et al., 1996) was employed to estimate the statistical power under these sample sizes.

### Procedure and instruments

The VAL-U scale was developed after several approaches. First, qualitative data obtained through focus groups and exploratory research methodologies were used for the first phase on item identification. Content validity for the VAL-U scale was developed by the assessment of 11 experts. A total of 20 Likert items (from 1 to 5 points) were considered after this process.

Secondly, two scales were used to test convergent and divergent validity. As civic values such as 1 solidarity, commitment-responsibility and respect-dialogue are expected from the qualitative part, resilience and social support were selected for convergent and divergent validity. All three are related to social support, However, Resilience can be considered as a mixed component (Solà-Sales et al., 2021) related to personality traits. In this way, only a relationship with commitment-responsibility would be expected.

The Brief Resilient Coping Scale (BRCS) was used to test validity (Sinclair & Wallston, 2004). The BCRS is a tool with adequate levels of reliability and validity. The original scale consists of 4 items and a single factor or dimension, with an index of internal consistency of *α* = .69 and test-retest reliability of .71. It was adapted to the Spanish population in the previous literature (Moret-Tatay et al., 2015). Since it measures resilience, it was included to test lack of relation with some subscales of VAL-U. Finally, the Social Support Survey Scale (MOS-SSS) was also included. It was developed by (Sherbourne & Stewart, 1991) and adapted to the Spanish population (Forns et al., 2005). It is divided into emotional support, material support and social relationships. In relation to their content, the internal consistency ranged from .92 to .83. As previously mentioned, it was included in order to test lack of relation with some subscales of VAL-U.

### Analysis

The analyses were developed through SPSS 22 and Amos 18.0. To examine the adequacy of indebtedness for the Spanish population in terms of psychometric properties, an exploratory factor analysis (EFA) was conducted. Assumptions were checked to ensure the application of factor analysis, such as high sample size, multivariate normality, linearity, and correlation between variables. Moreover, to find the suitable number of factors, Cattel’s scree-sediment graph as well as the Kaiser. In this way, the internal consistency of the scale was evaluated through Cronbach Alpha and McDonald’s Omega; items of homogeneity; KMO index and the Bartlett test of Sphericity. After removing the factorial solution proceeded to the completion of confirmatory factor analysis (CFA) though an independent sample, accompanied by the goodness of fit indices. No rotation of the data was used. Confirmation of the adequacy of the model has been used within the absolute fit indices; the chi-square statistic *X*^2^, and its ratio among degrees of freedom where values under 2 are recommendable. In terms of incremental fit indices, the comparative fit index (CFI), was selected. This follows a range of values between 0 and 1 and the reference value is .90. Finally, within parsimony adjustment indices, the root mean square error approximation (RMSEA) of the RMSR. Similarly, the smaller its value, the better the fit, the reference value being .05.

### Ethics

The university’s ethics committee’s approval of the study was obtained, ensuring that the principles of the Helsinki declaration were followed. University students were contacted to participate in the study with prior informed consent.

## Results

Cronbach’s alpha was = .675, and the percentage of total variance explained of 40.47%. Table 1 depicts the descriptive analysis, Cronbach’s alpha, kurtosis, skewness, and exploratory factor loadings between items.

**Table 1:** Means, standard deviation (SD), kurtosis, skewness and exploratory factor loadings (*n* = 162)

	Mean	SD	Skewness	Kurtosis	Cronbach alpha if deleted
Item 1	2.49	1.06	0.30	− 0.73	.655
Item 2	3.60	1.09	− 0.70	0.11	.663
Item 3	4.10	0.92	− 1.19	1.74	.651
Item 4	1.52	0.83	1.95	4.17	.711
Item 5	2.34	0.91	0.08	− 0.81	.655
Item 6	3.22	1.23	− 0.27	− 0.87	.666
Item 7	3.35	0.93	− 0.41	− 0.16	.644
Item 8	2.44	0.96	0.37	− 0.30	.655
Item 9	4.37	0.70	− 0.76	− 0.15	.655
Item 10	4.41	0.68	− 1.32	3.33	.656
Item 11	4.22	0.70	− 0.99	2.40	.666
Item 12	4.11	0.78	− 0.83	1.13	.652
Item 13	4.22	0.66	− 0.52	0.47	.665
Item 14	1.62	0.77	1.09	0.62	.697
Item 15	4.14	0.65	− 0.27	− 0.05	.677
Item 16	2.87	0.95	− 0.09	− 0.50	.642
Item 17	3.81	0.78	− 0.22	− 0.34	.658
Item 18	4.35	0.78	− 1.01	0.37	.660
Item 19	4.07	0.72	− 0.72	1.54	.660
Item 20	2.91	1.06	− 0.14	− 0.74	.674

**Fig. 1:**
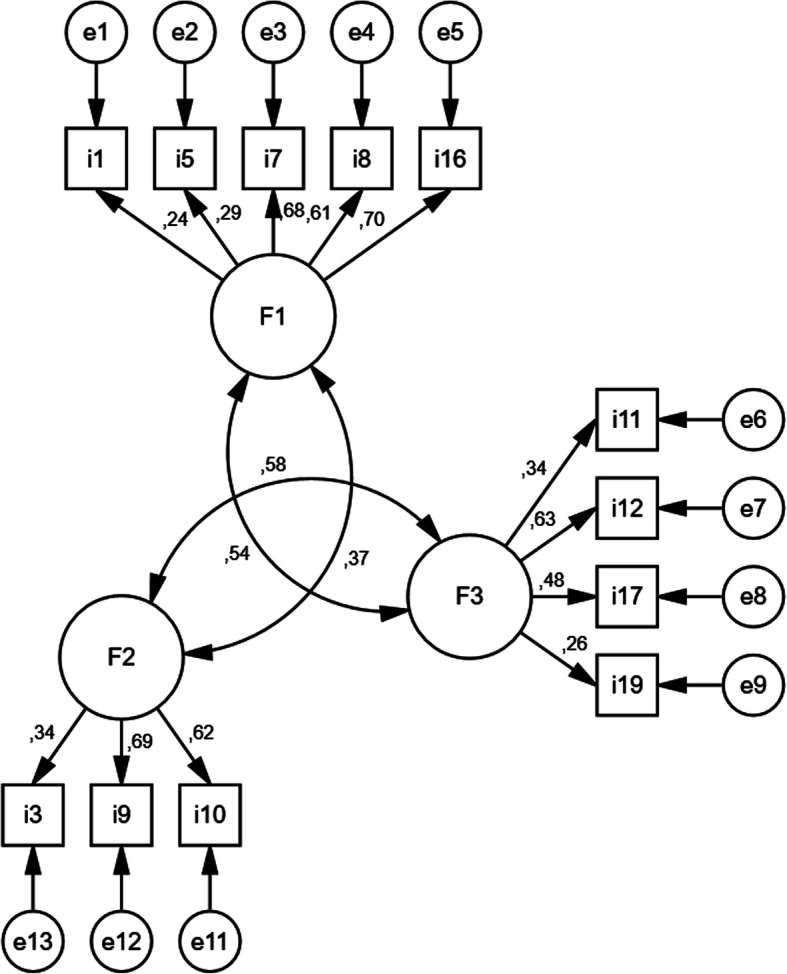
Factor loading in a 3-factor solution under the ML method (*n* =228)

With regard to the Exploratory Factor Analysis (EFA) in the first subsample, the Bartlett’s test of sphericity reached a *p* <.001 with a chi-square of 645.61 (df = 190) and the sample index value of Kaiser-Meyer-Olkin (KMO) was 0.70. The scree-test recommended three-factor solution. Table 2 characterizes the factor loadings, were values over 0.40 were included in accordance with previous literature (Constant et al. 2008). Even if several methods can be chosen, the maximum likelihood (ML) method seems to be the primary estimation procedure in the field. Moreover, no missing data was identified in the current sample to employ other interesting method to address this kind of issues. However, as the DWLS estimator seems to be ideal for handling ordinal data, this was also employed (Li, 2016). In this way, the Confirmatory Factor Analysis (CFA) confirmed, through an independent sample, a three-factor solution. The model presented an optimal fit for ML. The goodness of fit indices global scale was *X*^2^ /df=1.58, CFI = .90, IFI = .915, and RMSEA = .02. For the DWLS method the CFA depicted not such an optimal result but acceptable: *X*^2^/df=1.87; CFI = .88, IFI = .88, and RMSEA = .07. In order to summarise the information of each factor, these were labelled as follows: (i) factor 1, commitment (items 1, 5, 7, 8 and 16) and the Cronbach’s alpha as well as the McDonald’s Omega coefficient were *α*. = .68 and *ω* = .68; (ii) factor 2, commitment-responsibility (items 3, 9 and 10) and *α*. = .64 and *ω* = .65; and (iii) factor 3, respect-dialogue and *α*. = .68 and *ω* = .64 (items 11. 12, 17 and 19). Even if these values were not optimal, they were considered because of their holistical value (Constant et al., 2018). Hierarchical Factorial validity (Beland et al., 2017; Gignac et al., 2019) was also addressed though the average calculation of each subscale, but the second order solution depicted poor vales *α*. = .44, *ω* hierarchical (0.44), in comparison with the *ω* total (0.69), following as example this procedure from prior literature (Fung, Chow, & Cheung, 2020). To test the criterion validity of the scale, this was correlated between the resilience and social support, as depicted in Table 3. Finally, Fig. 1 shows the final factor structure, in terms of factor loading. In order to check the stability of the proposed tool, a correlation between test and retest (*n*=20 shows a high level of time stability rho (19) = 0.56; *p* < 0.001.

**Table 2:** Factor loading for 3-factor solution (*n* =228)

	Model 1		
	1	2	3
Item 1	**.411**	− .037	.232
Item 2	.393	.013	.272
Item 3	.215	.221	**.565**
Item 4	−.173	−.313	−.261
Item 5	**.400**	.130	.192
Item 6	.150	.155	.347
Item 7	**.754**	.226	.110
Item 8	**.631**	.129	−.045
Item 9	.047	.500	**.619**
Item 10	.080	.436	**.655**
Item 11	.003	**.639**	.225
Item 12	.171	**.595**	.334
Item 13	.168	.369	.242
Item 14	−.068	−.366	−.060
Item 15	−.065	.359	.255
Item 16	**.588**	.305	.156
Item 17	.221	**.425**	.218
Item 18	.287	.142	.155
Item 19	.130	**.446**	.308
Item 20	.239	−.027	.043

**Table 3:** Pearson coefficients among Factors and construct validity (*n* =228)

	F1	F2	F3	Resilience	Emotional support (ES)	Material support (MS)	Social relationships (SR)	Affective support (AS)
F1	1		.					
F2	.207**	1	.					
F2	.194*	.401**	1					
Resilience	.176*	.298**	− .001	1				
ES	− .137	.204**	.145	.131	1			
MS	− .123	.212**	.158*	.049	.882**	1		
SR	.515**	.413**	.178*	.258**	.019	.021	1	
AS	.258**	.223**	.463**	− .021	− .052	− .069	.214**	1

*p<.05; **p<.01

## Discussion

The objective of the research was to develop a measurement tool that makes it possible to assess changes related to SL experiences. The five selected factors - dialogue, responsibility, solidarity, commitment, and respect - are reflected in this instrument, although in the end they have been grouped into 3 factors: factor 1 solidarity, factor 2 commitment-responsibility and factor 3 respect-dialogue.

SL makes it possible to develop ecological values related to citizenship and are of interest in promoting social justice and democratic values. In this way, pedagogical methods and techniques that promote critical reflection, structured discussion and social justice are of interest for future generations. In addition, SL in class can also promote more positive attitudes about pressing social problems. Therefore, it is of interest that future research explores not only how the students can face social problems of interest, but also how SL influences attitudes (Ruiz-Ordóñez et al., 2020).

One of the possible explanations for the relationships found between the sub-factors, or lack thereof, with resilience could be the naturalness of resilience. According to recent authors, resilience could be defined as a mixed component of personality (Solà-Sales et al., 2021), which would support its relationship with more internal variables such as that described in factor two. On the other hand, social support would be found in all civic values factors. In this case, a strong relationship has been found with the subscale Social Relationships subscale in MOS, while other subscales related to emotional aspects have shown no relationship, supporting this explanation. In this scenario, it is of interest to know the perceptions that the students have of SL to consider the effectiveness of the teaching-learning processes. Moreover, one should bear in mind that SL is a methodology that is based on the detection of needs and social change, and this occurs because there is an intervention in the real context that transcends the university classroom itself. Therefore, any to measurement tool that helps us to better understand the role of SL in our University students is twofold, since it implies both applied and theoretical levels of relevance. Some of the practical implications are to know and measure the impact that the SL methodology has on university students in relation to values linked to civic commitment. Scores were grouped into 3 factors: solidarity, commitment-responsibility, and respect-dialogue.

Having a vision about civic engagement is essential to understand the experiences of students in Service-Learning and to develop or implement current theoretical models. Although current research has also recognised socio-cognitive components such as values, skills, and knowledge. We expect that these aspects will influence the understanding of education and civic engagement. This manuscript has one main limitation, namely that the sample was selected through non-probability sampling, which can introduce distortions into the results when one considers that the final sample may have a high component of self. Furthermore, finding acceptable but not-optimal values for internal consistency makes it imperative to develop direct and systematic replications of the new instrument. Future lines of research might consider the role of age and differences regarding the benefits of SL in students who are older. Older students may value more the integration of practical learning in the classroom, so that they more easily recognise communities where the connection between classroom and reality can be more adequately made. However, age is a variable that is rarely taken into account in SL surveys, since younger and older students benefit equally from the benefits of this methodology (Dorsey, 2001; Freeman & King, 2001).

## Conclusions

The objective of this work was to develop a Values and Civic Attitudes Scale for University Students’ Service-Learning assessment instrument. After a qualitative approach in the development of the Val-U scale, a factorial solution of three constructs was found, and acceptable psychometric properties were concluded, both at the exploratory and confirmatory levels. The new instrument showed convergence and divergence with other scales in the field of resilience and social support. Therefore, we can conclude that this is a good tool for the measurement of the construct under study in the university population involved in service-learning.

## References

[CR1] Beland S, Cousineau D, Loye N (2017). Using the Mcdonald’s omega coefficient instead of Cronbach’s alpha. Mcgill Journal of Education.

[CR2] Burton, S. L. (2018). *Engaged scholarship and civic responsibility in higher education: IGI Global.* 10.4018/978-1-5225-3649-9.

[CR3] Caprara GV, Kanacri BPL, Gerbino M, Zuffianò A, Alessandri G, Vecchio G, Caprara E, Pastorelli C, Bridglall B (2014). Positive effects of promoting prosocial behavior in early adolescence: Evidence from a school-based intervention. International Journal of Behavioral Development.

[CR4] Chow SL, Fu KW, Ng YL (2020). Development of the hong kong identity scale: Differentiation between hong kong ‘locals’ and mainland Chinese in cultural and civic domains. Journal of Contemporary China.

[CR5] Constant HM, Moret-Tatay C, Benchaya MC, Oliveira MDS, Barros HM, Ferigolo M (2018). CBI-20: Psychometric properties for the Coping Behaviors Inventory for alcohol abuse in Brazil. Frontiers in psychiatry.

[CR6] Delano-Oriaran, O. O., Penick-Parks, M. W., & Fondrie, S. (Eds.). (2018). *Culturally Engaging Service-Learning With Diverse Communities:* IGI Global. 10.4018/978-1-5225-2900-2

[CR7] Dias D, Soares D (2018). Civic learning outcomes: a step towards an inclusive higher education. International Journal of Inclusive Education.

[CR8] Dienhart C, Maruyama G, Snyder M, Furco A, McKay MS, Hirt L, Huesman R (2016). The impacts of mandatory service on students in service-learning classes. The Journal of Social Psychology.

[CR9] Dorsey B (2001). Linking Theories of Service-Learning and Undergraduate Geography Education. Journal of Geography.

[CR10] Erdfelder E, Faul F, Buchner A (1996). GPOWER: A general power analysis program. Behavior research methods, instruments, & computers.

[CR11] Forns M, Amador JA, Kirchner T, Gómez J, Muro P, Martorell B (2005). Psychometric Properties of the Spanish Version of the Moos Coping Response Inventory for Youth. Psychological Reports.

[CR12] Freeman NK, King S (2001). No title found. Early Childhood Education Journal.

[CR13] Fung S, Chow EO, Cheung C (2020). Development and Evaluation of the Psychometric Properties of a Brief Wisdom Development Scale. International Journal of Environmental Research and Public Health.

[CR14] Gerbino M, Zuffianò A, Eisenberg N, Castellani V, Luengo Kanacri BP, Pastorelli C, Caprara GV (2018). Adolescents’ Prosocial Behavior Predicts Good Grades Beyond Intelligence and Personality Traits. Journal of Personality.

[CR15] Gignac GE, Reynolds MR, Kovacs K (2019). Digit Span Subscale Scores May Be Insufficiently Reliable for Clinical Interpretation: Distinguishing Between Stratified Coefficient Alpha and Omega Hierarchical. Assessment.

[CR16] Hébert A, Hauf P (2015). Student learning through service learning: Effects on academic development, civic responsibility, interpersonal skills and practical skills. Active Learning in Higher Education.

[CR17] Huppert FA (2009). Psychological Well-being: Evidence Regarding its Causes and Consequences. Applied Psychology: Health and Well-Being.

[CR18] Jaeger AJ, Thornton CH (2006). Neither Honor nor Compensation: Faculty and Public Service. Educational Policy.

[CR19] Li CH (2016). Confirmatory factor analysis with ordinal data: Comparing robust maximum likelihood and diagonally weighted least squares. Behavior Research Methods.

[CR20] Martínez-Campillo A, Sierra-Fernández MD, Fernández-Santos Y. (2019). Service-Learning for Sustainability Entrepreneurship in Rural Areas: What Is Its Global Impact on Business University Students? *Sustainability*, *11*(19), 5296. https://doi.org/10.3390/su11195296

[CR21] Moret-Tatay C, Fernández-Muñoz JJ, Civera-Mollá C, Navarro-Pardo E, Alcover-de-la-Hera C (2015). Psychometric properties and Factor structure of the BRCS in an elderly Spanish sample. Anales de Psicología.

[CR22] Norman, Z. (2018). Understanding the Effect of Service-Learning Experiences on Students’ Cultural Competence in Higher Education. *SSRN Electronic Journal.*10.2139/ssrn.3167701.

[CR23] Prentice M (2007). Service learning and civic engagement. Academic Questions.

[CR24] Ruiz-Ordóñez Y, Salcedo-Mateu A, Turbi-Pinazo ÁM, Novella-García C, Moret-Tatay C (2020). CASD-14: A Questionnaire on Civic Attitudes and Sustainable Development Values for Service-Learning in Early Adolescents. Sustainability.

[CR25] Sanders MJ, Van Oss T, McGeary S (2016). Analyzing Reflections in Service Learning to Promote Personal Growth and Community Self-Efficacy. Journal of Experiential Education.

[CR26] Sherbourne CD, Stewart AL (1991). The MOS social support survey. Social Science & Medicine.

[CR27] Sinclair VG, Wallston KA (2004). The Development and Psychometric Evaluation of the Brief Resilient Coping Scale. Assessment.

[CR28] Solà-Sales, S., Pérez-González, N., Van Hoey, J., Iborra-Marmolejo, I., Beneyto-Arrojo, M. J., & Moret-Tatay, C. (2021). The Role of Resilience for Migrants and Refugees' Mental Health in Times of COVID-19. Healthcare (Basel, Switzerland), *9*(9). 10.3390/healthcare9091131 PMC8470365 34574904

[CR29] Weber PS, Weber JE, Sleeper BR, Schneider KL (2004). Self-efficacy toward service, civic participation and the business student: Scale development and validation. Journal of business ethics.

